# Supplementary material for: TFEB/Mitf links impaired nuclear import to autophagolysosomal dysfunction in C9-ALS
Source: eLife. 2020 Dec 10;9:e59419. doi: 10.7554/eLife.59419 (PMC7758070; doi:10.7554/eLife.59419)
Supplement: Supplementary file 1. — Flies expressing 30 G4C2 repeats in the eye under control of GMR-GAL4 were crossed to the indicated UAS line and scored for enhancement (<0) or suppression (>0) as described (Zhang et al., 2015a). [file elife-59419-supp1.docx]

**Supplementary File 1.** **Candidate Screen of autophagy-related genes.**

| **Drosophila stock** | **Effect on gene** | **Eye Score** |
| --- | --- | --- |
| *UAS-Atg6^RNAi^* | RNAi line | -3.5 |
| *UAS-Atg18a^RNAi^* | RNAi line | -3 |
| *UAS-Atg1* | cDNA overexpression | -3 |
| *UAS-Atg7* | cDNA overexpression | -2.5 |
| *UAS-Atg101^RNAi^* | RNAi line | -2 |
| *UAS-Atg8a^RNAi^* | RNAi line | -2 |
| *UAS-Atg5^RNAi^* | RNAi line | -2 |
| *UAS-Atg6^RNAi^* | RNAi line | -2 |
| *UAS-Atg8a^RNAi^* | RNAi line | -2 |
| *UAS-Ref(2)P^RNAi^* | RNAi line | -2 |
| *UAS-Atg14^RNAi^* | RNAi line | -2 |
| *UAS-Atg16^RNAi^* | RNAi line | -1.5 |
| *UAS-Atg16^RNAi^* | RNAi line | -1.5 |
| *UAS-Atg17^RNAi^* | RNAi line | -1.5 |
| *UAS-Atg6^RNAi^* | RNAi line | -1 |
| *UAS-Atg8b^RNAi^* | RNAi line | -1 |
| *UAS-bchs^RNAi^* | RNAi line | -1 |
| *UAS-Atg8b^RNAi^* | RNAi line | -1 |
| *UAS-Atg9^RNAi^* | RNAi line | -0.5 |
| *UAS-Atg18a^RNAi^* | RNAi line | -0.5 |
| *UAS-Gyf^RNAi^* | RNAi line | -0.5 |
| *Atg6^00096^* | P-element insertion | 0 |
| *UAS-Atg4b^RNAi^* | RNAi line | 0 |
| *UAS-Atg17^EP^* | EP overexpression | 0 |
| *bchs^58^* | EMS mutagenesis | 0 |
| *UAS-Atg4a^RNAi^* | RNAi line | 0 |
| *UAS-Atg4a^RNAi^* | RNAi line | 0 |
| *UAS-Atg10^RNAi^* | RNAi line | 0 |
| *UAS-Atg16^RNAi^* | RNAi line | 0 |
| *UAS-Atg9^RNAi^* | RNAi line | 0 |
| *UAS-lt^RNAi^* | RNAi line | 0 |
| *UAS-Atg7^RNAi^* | RNAi line | 0 |
| *Atg7^d06996^* | P-element insertion | 0 |
| *UAS-Atg4a^RNAi^* | RNAi line | 1 |
| *UAS-Atg8a^RNAi^* | RNAi line | 1 |
| *Bchs^17^* | EMS mutagenesis | 1 |
| *UAS-Atg8a^EP^* | EP overexpression | 1 |
| *UAS-Atg2^EP^* | EP overexpression | 1 |
| *UAS-Atg7^RNAi^* | RNAi line | 1 |
| *UAS-Atg13^RNAi^* | RNAi line | 1.5 |
| *UAS-Atg14^RNAi^* | RNAi line | 1.5 |
| *UAS-Atg3^EP^* | EP overexpression | 2 |
| *UAS-Atg18b^RNAi^* | RNAi line | 2 |
| *UAS-Atg2^RNAi^* | RNAi line | 2 |
| *Snap29^B6-21^* | EMS | 2 |
| *UAS-Atg3^RNAi^* | RNAi line | 3 |
| *UAS-Atg2^RNAi^* | RNAi line | 3 |
| *UAS-Atg18b^RNAi^* | RNAi line | 3 |
| *Atg4b^P0997^* | P-element insertion | 4 |
| *UAS-bchs-HA* | cDNA overexpression | 4 |
