# Supplementary material for: TFEB/Mitf links impaired nuclear import to autophagolysosomal dysfunction in C9-ALS
Source: eLife. 2020 Dec 10;9:e59419. doi: 10.7554/eLife.59419 (PMC7758070; doi:10.7554/eLife.59419)
Supplement: Supplementary file 2. — Motor cortex from postmortem brain autopsies from four C9-ALS patients and four non-neurological disease controls were used is this study (Figure 7). Patient ID, cause of death (diagnosis), gender, age at death, post-mortem interval (PMI) in hours, and presence of C9orf72 expanded hexanucleotide repeat are indicated. [file elife-59419-supp2.docx]

**Supplementary File 2. Demographics of human patients.**

|  | Patient ID | Diagnosis | Gender | Age at death | PMI (hrs) | C9orf72 mutation |
| --- | --- | --- | --- | --- | --- | --- |
| Control | 70 | Multiple medical problems | Female | 59 | 7 | No |
|  | 91181 | Cardiac arrest | Male | 53 | 12 | No |
|  | 91268 | Cardiac failure | Male | 76 | 12 | No |
|  | 73 | Cardiovascular failure | Male | 74 | 4 | No |
| C9-ALS | 88 | FTD/ALS | Male | 59 | 10 | Yes |
|  | 67 | Familial ALS | Male | 47 | 5 | Yes |
|  | 1629 | FTD/ALS | Female | 55 | NA | Yes |
|  | 473111 | ALS | Male | 47 | NA | Yes |
